# Evaluation of non-alcoholic fatty liver disease in patients with inflammatory bowel disease using controlled attenuation parameter technology: A Taiwanese retrospective cohort study

**DOI:** 10.1371/journal.pone.0252286

**Published:** 2021-05-27

**Authors:** Hsu-Heng Yen, Pei-Yuan Su, Siou-Ping Huang, Lisha Wu, Tsui-Chun Hsu, Ya-Huei Zeng, Yang-Yuan Chen

**Affiliations:** 1 Division of Gastroenterology, Department of Internal Medicine, Changhua Christian Hospital, Changhua, Taiwan; 2 Department of Electrical Engineering, Chung Yuan Christian University, Taoyuan, Taiwan; 3 General Education Center, Chienkuo Technology University, Changhua, Taiwan; 4 Taiwan Association for the Study of Small Intestinal Diseases (TASSID), Taipei, Taiwan; 5 Department of Hospitality Management, MingDao University, Changhua, Taiwan; Kaohsiung Medical University, TAIWAN

## Abstract

**Background/purpose:**

An increased prevalence of non-alcoholic fatty liver disease (NAFLD) is observed in patients with inflammatory bowel disease (IBD) in Western countries. Both intestinal inflammation and metabolic factors contribute to the pathogenesis of IBD-associated NAFLD. The burden of NAFLD is not clear in the Asian population. This study aimed to evaluate the prevalence of NAFLD and liver fibrosis in a cohort of Taiwanese patients with IBD.

**Methods:**

From January to December 2019, patients with IBD who underwent ultrasound examination were enrolled. Hepatic steatosis and fibrosis were measured with liver stiffness measurement (LSM) and controlled attenuation parameter (CAP) using FibroScan. Patients with a history of excessive alcohol or recent steroid use were excluded. Univariate and multivariate analysis were performed.

**Results:**

A total of 81 consecutive patients were enrolled and included in the analysis (45 with ulcerative colitis, 36 with Crohn’s disease). The median age was 42 years old. The patients were classified in terms of body mass index as normal weight (54.3%), underweight (11.1%), overweight (28.4%), and obese (6.2%). The mean CAP increased to 162.22 dB/m in the underweight group, 210.86 dB/m in the normal weight group, 260.7 dB/m in the overweight group, and 274.0 dB/m in the obese group. NAFLD was observed in 29.6% of the patients, 1.2% of which had significant fibrosis. Increased body mass index (odds ratio [OR] 1.33, 95% confidence interval [CI] 1.1–1.62) and older age at IBD diagnosis (OR: 1.05, 95% CI 1–1.11) was found to be associated with the presence of NAFLD.

**Conclusion:**

In this study, the prevalence of NAFLD was lower (29.6%) in IBD patients than in the Western population. Higher BMI and older age were associated with NAFLD in our study.

## Introduction

Inflammatory bowel disease (IBD, including ulcerative colitis [UC] and Crohn’s disease [CD]) is a chronic inflammatory disease affecting the gastrointestinal tract that has seen increased incidence and prevalence in Asia in the 21st century [1–4]. Non-alcoholic fatty liver disease (NAFLD) is becoming the most common chronic liver disease in the world, surpassing chronic hepatitis B [5] and hepatitis C [6] and affecting 22.28%–51.04% of the Asian population [7]. IBD and NAFLD were traditionally Western diseases and are now being increasingly observed in the Asian population owing to Westernization.

Non-alcoholic fatty liver disease had been rarely described in patients with IBD [8] before the introduction of modern treatments for the disease. These patients had been traditionally viewed as underweight and/or malnourished due to uncontrolled bowel inflammation. Now, with the advent of more effective therapy, these patients may become more overweight and obese compared with those of the general population [9–11]. Consequently, these patients are at increased risk of developing NAFLD [8] and the subsequent risk of developing liver cirrhosis and hepatocellular carcinoma [7], especially those with concurrent use of hepatotoxic agents for IBD. In a recent meta-analysis of 27 studies, the prevalence of NAFLD among IBD patients was found to be statistically significantly higher than that in the general population (32% vs 25.2%; P < 0.001) [9]. However, the majority of the study involves reports from Western countries and only one article described the presence of NAFLD in 21.8% of CD patients from Japan [12].

Transient elastography (TE) is a non-invasive test that is quick and easy to perform, with high accuracy and reproducibility in detecting advanced fibrosis and cirrhosis [11, 13–15]. The controlled attenuation parameter (CAP) is the measurement of the degree of ultrasound attenuation caused by hepatic fat at the central frequency of the FibroScan [11, 13–15]. The simultaneous measurement of TE and CAP is currently feasible in one machine and helps clinicians choose the treatment for various chronic liver diseases [6, 14, 16, 17]. To date, only a few studies have focused on concurrent IBD and NAFLD in the Asian population [9, 12]. The aim of our study was to investigate the prevalence of and the factors associated with NAFLD in an Asian population with IBD.

## Materials and methods

The medical records of patients diagnosed with IBD at Changhua Christian Hospital, Taiwan, were retrospectively reviewed from January 2019 to December 2019. From January 2018, patients diagnosed with IBD, including UC and CD, had received integrated hospital care from a trained IBD nurse (Ms. Hsu TC). Patients received at least one annual laboratory examination for disease monitoring. From January 2019, the in-hospital screening program for liver disease for patients with IBD included abdominal ultrasound screening and liver stiffness measurements (LSM) with the CAP for the screening of liver fibrosis and steatosis. The LSMs and CAP measurements were performed using FibroScan (Echosens) by one experienced operator (Ms. Wu L) who has had more than 25 years’ experience of ultrasound examination and had performed more than 5000 FibroScan examinations [11, 14]. All patients were evaluated using the 3.5 MHz standard M probe. Both the LSM and CAP value were obtained simultaneously. Ten LSMs were recorded, and the median value was calculated by the equipment as the final score. The following liver stiffness cutoff values were used for staging: F0, ≤6.5 kPa; F1, ≤8 kPa; F2, ≤9.5 kPa; F3, ≤12 kPa, and F4, >12 kPa [13, 18]. The diagnosis of NAFLD was defined as the median value of CAP ≥248 dB/m [13, 17]. The liver steatosis ultrasound readings were graded as normal, mild, moderate, and severe [19] by one operator (Ms. Wu L). Patients were classified according to BMI: underweight, BMI < 18.5; normal weight, 18.5 ≤ BMI < 25.0; overweight if 25.0 ≤ BMI < 30.0; and obese if BMI ≥ 30.0. Patients enrolled in the present study met the following inclusion criteria: (1) documented diagnosis of CD or UC for more than 6 months and (2) abdominal ultrasound and LSM and CAP examination during clinical remission. The exclusion criteria were as follows: (1) hazardous alcohol intake and (2) lack or failure of LSM examination or unreliable measurement. The following variables were extracted from medical records: age, sex, disease type, age at diagnosis, surgical history, laboratory measurements and comorbidities, including hypertension, diabetes, and dyslipidemia. The requirement for informed consent for data extraction was waived by the institutional review board because the study involved minimal risk. This study was approved by the institutional review board of Changhua Christian Hospital (approval number: CCH IRB 190814).

### Statistical analysis

The extracted data were organized using Microsoft Excel and analyzed using MedCalc Statistical Software version 19.16 (MedCalc Software bvba, Ostend, Belgium; https://www.medcalc.org; 2020). Continuous data were expressed as mean and standard deviation or as median and interquartile range (IQR) for normally and non-normally distributed data, respectively. Categorical variables were presented as numbers and percentages. The mean values with normally distributed variables were compared using the independent sample’s Student’s *t*-test. The Mann–Whitney U-test and Kruskal–Wallis test were used to compare the mean values of 2 and ≥3 groups of non-normally distributed variables, respectively. Multivariate logistic regression analysis was performed to identify factors associated with NAFLD. Selected variables with p-value<0.10 from crude mode for backward elimination procedure in the multivariable model. All *P*-values < 0.05 were considered statistically significant.

## Results

### Clinical features of patients with IBD

**Fig 1:**
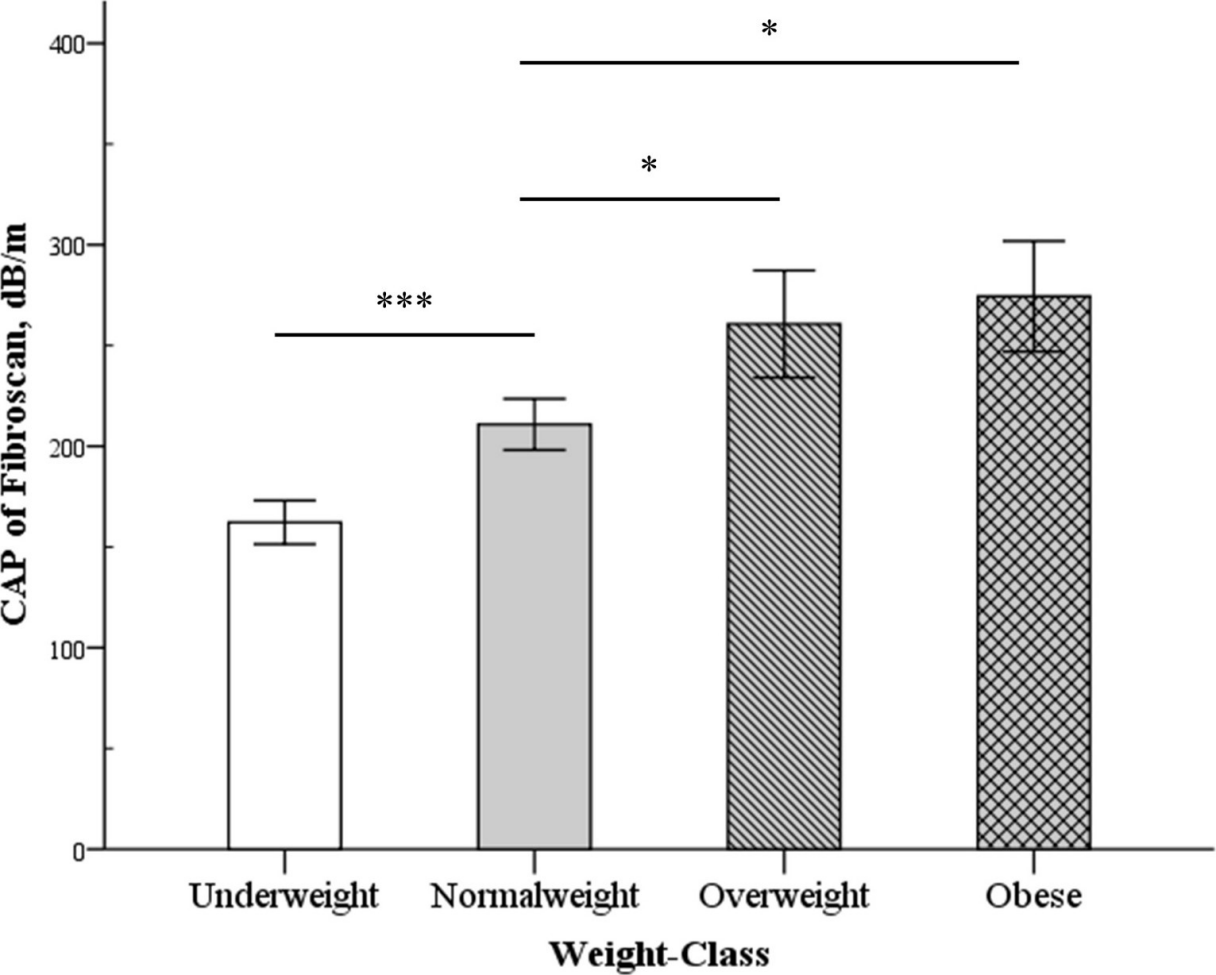
Differences in the FibroScan controlled attenuation parameter (CAP) scores (vertical line) between the different weight classes (horizontal line). Data are expressed as mean ± standard error. *P < 0.05 and ***P < 0.001.

**Fig 2:**
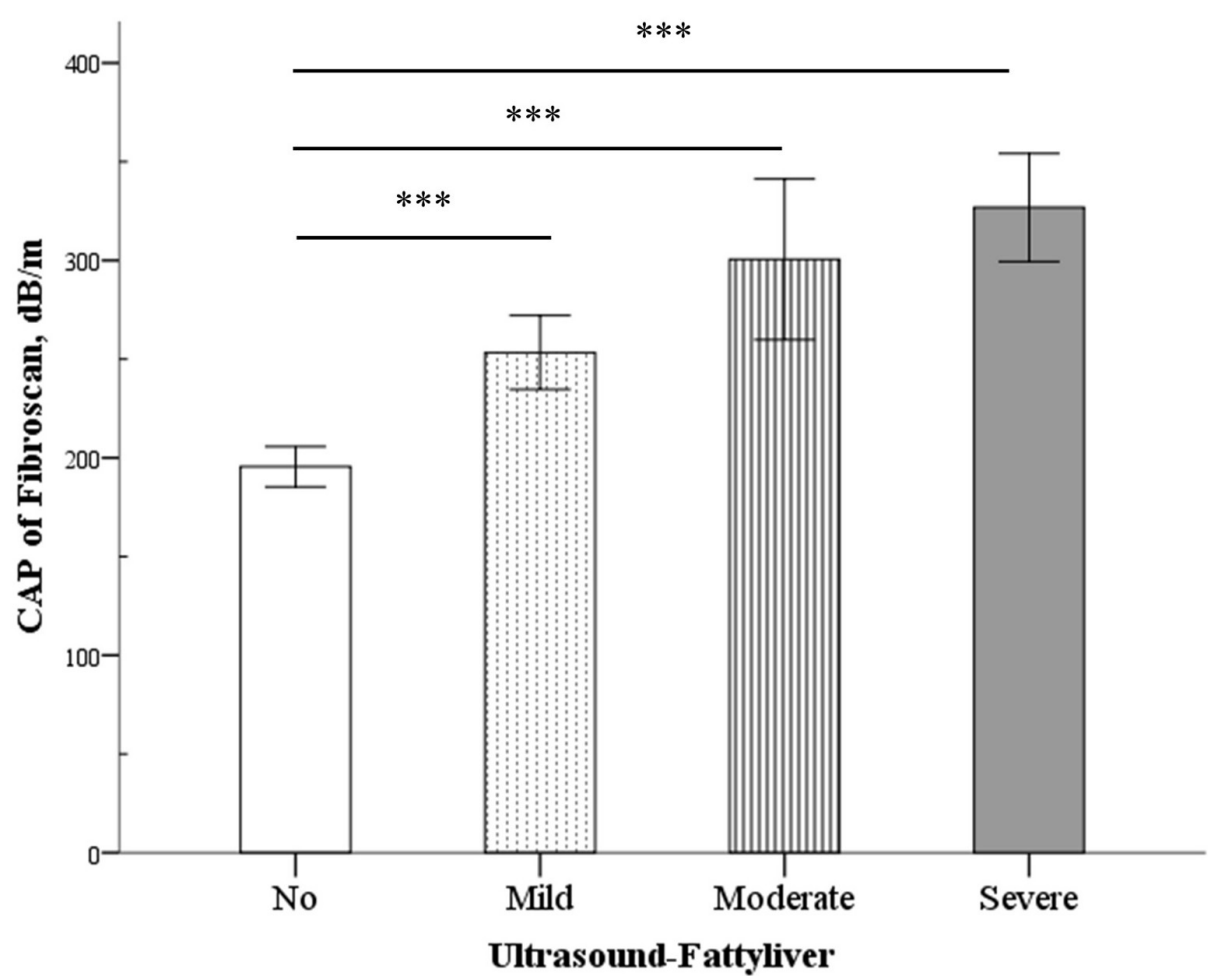
Differences in the FibroScan controlled attenuation parameter (CAP) scores (vertical line) between different ultrasound fatty (horizontal line) grades. Data are expressed as mean ± standard error. ***P < 0.001.

A total of 81 patients with IBD (CD/UC: 36/45) met the inclusion criteria. The clinical characteristics of all patients are presented in Table 1. The mean age of the patients was 43.54 years, and the majority were men (71.6%). The median disease duration was 4 years, and the median BMI was 22.41 kg/m^2^. Patients with CD had a high bowel resection rate (50% vs 2.2%, p < 0.001) and a higher proportion of biologic agent use compared with those with UC (66.7% vs 11.1%, p < 0.001). Significant liver fibrosis (estimated liver fibrosis ≥ F2) was found in 4.9% of patients, of which, 1.2% had liver cirrhosis. About 11.1% of patients were classified as underweight, and 6.2% were obese. The CAP value increased with increasing weight and severity of fatty change on ultrasound (Figs 1 and 2). The distribution of body weight based on CAP and LSM was similar between CD and UC patients. Gallstones were found in 9.9% of patients.

**Table 1:** Patient baseline characteristics.

	All Patients	CD	UC	
Number of cases	(n = 81)	(n = 36)	(n = 45)	P-value
**Age, year ± SD**	43.54 ± 14.42	40.33 ± 14.38	46.11 ± 14.08	0.073
**Gender (Male), n (%)**	58 (71.6%)	28 (77.8%)	30 (66.7%)	0.270
**BMI, kg/m**^**2**^	22.41 (20.31–25.56)	22.35 (19.52–25.76)	22.58 (20.83–25.51)	0.509
**IBD duration, year**	4 (3–8)	4 (3–7)	4 (2–10)	0.962
**Age at disease onset, year**	37.26 ± 13.16	34.58 ± 12.94	39.4 ± 13.08	0.102
**Bowel resection, n (%)**	19 (23.5%)	18 (50%)	1 (2.2%)	<0.001
**Weight class**				
**Underweight**	9 (11.1%)	5 (13.9%)	4 (8.9%)	0.501
**Normal weight**	44 (54.3%)	19 (52.8%)	25 (55.6%)	0.980
**Overweight**	23 (28.4%)	10 (27.8%)	13 (28.9%)	1.000
**Obese**	5 (6.2%)	2 (5.6%)	3 (6.7%)	1.000
**Hypertension**	5 (6.2%)	3 (8.3%)	2 (4.4%)	0.651
**Diabetes mellitus**	4 (4.9%)	3 (8.3%)	1 (2.2%)	0.318
**Hyperlipidemia**	1 (1.2%)	1 (2.8%)	0	0.444
**Use of anti-TNF**	29 (35.8%)	24 (66.7%)	5 (11.1%)	<0.001
**Use of anti-integrin**	6 (7.4%)	4 (11.1%)	2 (4.4%)	0.399
**Hepatitis B, n (%)**	8 (9.9%)	3 (8.3%)	5 (11.1%)	0.727
**Hepatitis C, n (%)**	2 (2.5%)	1 (2.8%)	1 (2.2%)	1.000
**Gallbladder stone, n (%)**	8 (9.9%)	6 (16.7%)	2 (4.4%)	0.130
**Steatosis on ultrasound**				
**No**	53 (65.4%)	22 (61.1%)	31 (68.9%)	0.620
**Mild**	17 (21%)	6 (16.7%)	11 (24.4%)	0.562
**Moderate/Severe**	11 (13.6%)	8 (2.2%)	3 (6.7%)	0.054
**WBC, ×10**^**3**^**/μL**	5.7 (4.9–7.2)	5.55 (4.85–7.2)	5.8 (4.9–7.5)	0.631
**Seg, %**	61.99 ± 11.07	61.99 ± 11.81	61.99 ± 10.58	1.000
**Lym, %**	28.4 (20.2–32.8)	28.05 (19.55–35.75)	29.9 (20.4–32.1)	0.820
**Hb, g/dL**	13.9 (12.7–14.6)	13.4 (12.15–14.6)	14.1 (12.9–14.7)	0.337
**Platelet, ×10**^**3**^**/μL**	266 (220–327)	256.5 (206–333)	268 (221–301)	0.604
**ESR**	11 (5–19)	11 (5.5–28)	9 (5–16)	0.345
**Albumin, g/dL**	4.4 (4–4.6)	4.3 (3.9–4.6)	4.4 (4.1–4.5)	0.248
**GOT, U/L**	23 (19–27)	24 (18–27.5)	22 (19–27)	0.801
**GPT, U/L**	16 (12–26)	18 (12–27)	16 (13–23)	0.962
**CRP, mg/dL**	0.1 (0.04–0.46)	0.11 (0.04–0.67)	0.1 (0.05–0.34)	0.665
**Creatinine, mg/dL**	0.83 ± 0.2	0.85 ± 0.18	0.81 ± 0.21	0.416
**eGFR, mL/min/1.73m^2**	100.77 ± 21.77	101.32 ± 22.9	100.33 ± 21.08	0.840
**FIB-4**	0.82 (0.56–1.29)	0.78 (0.45–1.22)	0.88 (0.62–1.43)	0.387
**Elasticity, kPa**	5.1 (4.2–5.8)	5 (4.05–5.7)	5.2 (4.3–5.8)	0.992
**CAP, dB/m**	223.53 ± 56.73	221.28 ± 67.18	225.33 ± 47.48	0.761
**NAFLD, CAP ≥ 248 dB/m**	24 (29.6%)	11 (30.6%)	13 (28.9%)	0.870
**LSM estimated fibrosis stage**				
**F0**	71 (87.7%)	31 (86.1%)	40 (88.9%)	0.745
**F1**	6 (7.4%)	2 (5.6%)	4 (8.9%)	0.688
**F2**	3 (3.7%)	2 (5.6%)	1 (2.2%)	0.582
**F4**	1 (1.2%)	1 (2.8%)	0	0.444

Data are expressed as n (%), median (interquartile range), or mean ± standard deviation. Categorical variables were compared using the χ^2^ test or the Fisher’s exact test; continuous variables were compared using the Student’s t-test or the Mann–Whitney U-test. CD, Crohn’s disease; UC, ulcerative colitis; BMI, body mass index; IBD, irritable bowel disease; TNF, tumor necrosis factor; WBC, white blood count; Seg,; Lym,; Hb, hemoglobin; ESR,; GOT,; GPT,; CRP, C-reactive protein; FIB-4,; eGFR, estimated glomerular filtration rate; CAP, controlled attenuation parameter; LSM,.

### Comparison of patients with and without NAFLD

In our cohort, the presence of NAFLD, defined as CAP ≥ 248 dB/m, was observed in 29.6% of patients (Table 2). Age, sex, disease type, history of bowel resection, and drugs used were similar between those with NAFLD and those without NAFLD. Compared with patients without NAFLD, those with NAFLD had higher BMI (25.85 vs 21.5, p < 0.001), shorter disease duration (3 vs 5 years, p = 0.028), older age at disease diagnosis (42.17 vs 35.19, p = 0.028), higher proportion of anti-integrin use (16.7% vs 1.8%, p = 0.025), higher GPT level (23 vs 15, p = 0.019), higher creatinine level (0.92 vs 0.79, p = 0.007). No patients in the underweight class were diagnosed with NAFLD in this cohort.

**Table 2:** Comparison of patient characteristics with and without NAFLD.

Variable	All Patients	NAFLD	No NAFLD	
**Case number**	(n = 81)	(n = 24)	(n = 57)	P-value
**Age, year**	43.54 ± 14.42	46.63 ± 12.44	42.25 ± 15.08	0.214
**Gender, male, %**	58 (71.6%)	18 (75%)	40 (70.2%)	0.660
**BMI, kg/m**^**2**^	22.41 (20.31–25.56)	25.85 (24.9–27.43)	21.5 (19.68–23.71)	<0.001
**Weight class**				
**Underweight**	9 (11.1%)	0	9 (15.8%)	0.052
**Normal weight**	44 (54.3%)	6 (25%)	38 (66.7%)	0.001
**Overweight**	23 (28.4%)	15 (62.5%)	8 (14%)	<0.001
**Obese**	5 (6.2%)	3 (12.5%)	2 (3.5%)	0.151
**Crohn’s disease**	36 (44.4%)	11 (45.8%)	25 (43.9%)	0.870
**IBD duration, year**	4 (3–8)	3.5 (2–5)	5 (3–10)	0.028
**Age at IBD diagnosis, year**	37.26 ± 13.16	42.17 ± 11.83	35.19 ± 13.23	0.028
**Bowel resection, n (%)**	19 (23.5%)	8 (33.3%)	11 (19.3%)	0.173
**Use of Biologic agent**	29 (35.8%)	10 (41.7%)	19 (33.3%)	0.475
**Use of anti-TNF**	6 (7.4%)	1 (4.2%)	5 (8.8%)	0.664
**Use of anti-integrin**	5 (6.2%)	4 (16.7%)	1 (1.8%)	0.025
**Diabetes mellitus**	4 (4.9%)	3 (12.5%)	1 (1.8%)	0.076
**Hyperlipidemia**	1 (1.2%)	1 (4.2%)	0	0.296
**Gallbladder stone, n (%)**	8 (9.9%)	4 (16.7%)	4 (7%)	0.228
**Steatosis on ultrasound**				
**No**	53 (65.4%)	4 (16.7%)	49 (86%)	<0.001
**Mild**	17 (21%)	10 (41.7%)	7 (12.3%)	0.008
**Moderate/Severe**	11 (13.6%)	10 (41.7%)	1 (1.8%)	<0.001
**WBC, ×10^3/μL**	5.7 (4.9–7.2)	5.6 (4.75–7.6)	5.7 (4.9–7.2)	0.698
**Seg, %**	61.99 ± 11.07	59.2 ± 10.89	63.17 ± 11.03	0.141
**Lym, %**	28.4 (20.2–32.8)	31.55 (23.35–36.85)	26.2 (19–31.7)	0.080
**Hb, g/dL**	13.9 (12.7–14.6)	14 (13.4–14.75)	13.4 (12.3–14.5)	0.145
**Platelet, ×10**^**3**^**/μL**	266 (220–327)	253 (197.5–285)	268 (220–330)	0.230
**ESR**	11 (5–19)	10 (3.5–15.5)	11 (5–22)	0.295
**Albumin, g/dL**	4.4 (4–4.6)	4.4 (4.2–4.6)	4.3 (4–4.5)	0.383
**GOT, U/L**	23 (19–27)	25.5 (18–30)	22 (19–26)	0.236
**GPT, U/L**	16 (12–26)	23 (15.5–30.5)	15 (12–23)	0.019
**CRP**	0.1 (0.04–0.46)	0.11 (0.05–0.36)	0.1 (0.04–0.59)	0.942
**Creatinine, mg/dL**	0.83 ± 0.2	0.92 ± 0.23	0.79 ± 0.17	0.007
**eGFR, mL/min/1.73m**^**2**^	100.77 ± 21.77	89.78 ± 19.9	105.4 ± 21	0.003
**Neutrophil-to-lymphocyte ratio**	2.2 (1.66–3.47)	1.77 (1.42–2.88)	2.45 (1.74–3.8)	0.080
**Elasticity, kPa**	5.1 (4.2–5.8)	5.25 (4.9–6.1)	5 (4–5.6)	0.158
**CAP, dB/m**	223.53 ± 56.73	294.46 ± 34.62	193.67 ± 32.26	<0.001
**LSE estimated fibrosis stage**				
**F0**	71 (87.7%)	19 (79.2%)	52 (91.2%)	0.152
**F1**	6 (7.4%)	3 (12.5%)	3 (5.3%)	0.354
**F2**	3 (3.7%)	1 (4.2%)	2 (3.5%)	1.000
**F4**	1 (1.2%)	1 (4.2%)	0	0.296

Data are expressed as n (%), median (interquartile range), or mean ± standard deviation. Categorical variables were compared using the χ^2^ test or the Fisher’s exact test; continuous variables were compared using the Student’s t-test or the Mann–Whitney U-test. NAFLD, non-alcoholic fatty liver disease; BMI, body mass index; IBD, irritable bowel disease; TNF, tumor necrosis factor; WBC, white blood count; Seg,; Lym,; Hb, hemoglobin; ESR,; GOT,; GPT,; CRP, C-reactive protein; eGFR, estimated glomerular filtration rate; CAP, controlled attenuation parameter; LSE,.

Table 3 shows the results of multivariate analyses for predictors of NAFLD. After adjustments, BMI (odds ratio [OR]: 1.33, 95% confidence interval [CI]: 1.1–1.62; p = 0.004) and age at IBD diagnosis (OR: 1.05, 95% CI: 1–1.11; p = 0.045) were the independent predictive factors of NAFLD.

**Table 3:** Multivariable analysis of factors associated with NAFLD.

Risk Factor	Univariate		Multivariate	
	crude OR (95% CI)	P-value	adjusted OR (95% CI)	P-value
**Age, y**	1.02 (0.99–1.06)	0.213	--	--
**Gender (Male)**	1.27 (0.43–3.77)	0.661	--	--
**BMI, kg/m**^**2**^	1.42 (1.18–1.72)	<0.001	1.33 (1.1–1.62)	0.004
**Crohn’s disease**	1.08 (0.42–2.82)	0.870	--	--
**IBD duration, year**	0.89 (0.79–1)	0.058	0.87 (0.74–1.03)	0.101
**Age at IBD diagnosis, year**	1.04 (1–1.08)	0.033	1.05 (1–1.11)	0.047
**Bowel resection**	2.09 (0.71–6.12)	0.178	--	--
**Use of anti-TNF**	1.43 (0.54–3.81)	0.476	--	--
**Use of anti-integrin**	0.45 (0.05–4.09)	0.480	--	--
**Hypertension**	11.2 (1.18–106.26)	0.035	--	--
**Diabetes mellitus**	8 (0.79–81.25)	0.079	--	--
**Hyperlipidemia**	4003568142 (0–0)	1.000	--	--
**Hepatitis B**	1.49 (0.33–6.78)	0.609	--	--
**Hepatitis C**	0 (0–0)	0.999	--	--
**Creatinine, mg/dL**	33.8 (2.18–525.09)	0.012	15.61 (0.51–475.19)	0.115

OR, odds ratio; CI, confidence interval; IBD, irritable bowel disease; TNF, tumor necrosis factor.

## Discussion

Our study reveals several important findings, namely, the prevalence of NAFLD and the identification of independent predictive factors for NAFLD in an Asian population with IBD. NAFLD was observed in 29.6% of our patients, 1.2% of which had significant fibrosis. Increased BMI and age at IBD diagnosis were found associated with the presence of NAFLD. To our knowledge, only a few studies reported the screening of NAFLD in Asian patients with IBD [9, 10, 20–22], and our study was the first report using CAP technology.

After hepatitis B vaccination and hepatitis C elimination programs [6, 23, 24] were introduced, NAFLD has become the most common chronic liver disease in the world [7, 25, 26], which poses the subsequent risk of progression to liver cirrhosis and the development of hepatocellular carcinoma. The prevalence of NAFLD has seen a rapid increase in the Asian population in the past decade [7, 25], with the highest prevalence in Iran (64.29%) and the lowest in Taiwan (30.79%) [7]. During the same period, the incidence of IBD has increased worldwide, particularly in the Asia-Pacific region [1, 2]. Our recent national cohort study in Taiwan reported a six-fold increase in the prevalence of IBD over the past 15 years. The importance of the co-existence of IBD and NAFLD is being increasingly recognized [8, 12, 20]. The prevalence of NAFLD among patients with IBD varied by the region and time period studied [8] in particular the advance of medical therapy in conjunction with larger societal trends in weight gain in the 21st century. The prevalence of NAFLD among patients with IBD ranges from 6% to 67% with a pooled overall prevalence from 19 studies that included 5620 patients with IBD of 27.5% [9, 12, 20]. An increased prevalence of NAFLD among patients with IBD compared with the general population was observed in North America (43% vs 24.1%) and Europe (31% vs 23.7%) [9, 27]. However, data regarding the epidemiology NAFLD among IBD patients from Asia are lacking. Shintaro et al. [12] reported an increased prevalence of NAFLD among 303 CD patients compared with the general population (21.8% vs 7.9%). Our present study is the first hospital-based study reporting the prevalence of NAFLD among patients with IBD that was comparable to previous reports on the general population in Taiwan [7]. The prevalence of NAFLD is low in our population (45.3%–71%) compared with that in Western countries [11, 13] as assessed using CAP. The prevalence rates of significant liver fibrosis (4.9%) in our cohort was lower than those reported for Western countries (12.2%) [11].

Both IBD and NAFLD involve a complex interplay between environmental and genetic factors. Chronic intestinal and hepatic inflammation may leads to the development of atherosclerosis and subsequent cardiovascular diseases [28]. Chiara et al. [11] reported NAFLD/IBD patients had increased cardiovascular and chronic renal diseases than those without NAFLD. Understanding the underlying causes and predisposing factors of NAFLD among patients with IBD is important to designing therapeutic strategies and improving patient prognosis [9, 14, 29]. The risk of developing NAFLD in the general population is related to diabetes, dyslipidemia, and obesity [7, 21]. In patients with IBD, disease-related factors, such as disease duration (mean difference, 1.59 years; 95% CI, 0.66–2.54) and previous intestinal surgery (odds ratio [OR] 1.39; 95% CI, 1.01–1.93), were proposed as precipitating factors for the development of NAFLD [9, 20]. The role of steroids and immunosuppressive medications remains controversial [9, 20, 30]. Only older age at IBD diagnosis and increased BMI were found as the independent predictive factors of NAFLD in our study. This may reflect the different genetic and environmental risk factors of NAFLD/IBD between the Eastern and Western populations [2, 7, 31, 32].

As NAFLD is mostly clinically silent, the recent guidelines did not address the specific assessment of NAFLD in the population with IBD [3, 4, 33]. The current gold standard for classifying the severity of chronic liver disease relies on liver biopsy specimens. The present study utilized TE with CAP technology that was available for the treatment evaluation of patients with chronic hepatitis B or C [6, 17, 18]. The procedure is non-invasive and provides quantitative information compared with abdominal ultrasonography, which can only grade patients for fatty liver status. In addition, TE with CAP is cost-effective and more widely available compared with MRI [34, 35]. The TE techniques had been used previously to evaluate IBD patients treated with methotrexate for screening and follow-up of liver fibrosis [36]. As intestinal ultrasound for is increasingly used for monitoring IBD disease activity [37], TE with P technology is feasible to be performed in the same unit as point-of-care service. We believe such evaluation may be helpful in improving IBD patient care, although the optimal interval and benefits are yet to be studied.

The study had several limitations. First, the study size is limited due to the low prevalence rate of IBD in Taiwan [38]. Our study was conducted retrospectively at a single medical center; therefore, we may have included patients with more severe IBD requiring therapy in the tertiary care unit. Some patients were referred from primary care units, we were unable to assess the cumulative steroid dosage in the clinical history of all patients in the present study. Second, the current study includes patients with other liver diseases (e.g. chronic viral hepatitis). This limits adequate distinction between fatty liver due to metabolic syndrome and obesity versus drug-induced liver injury and drug-induced hepatic steatosis[39, 40]. Third, the Taiwan national insurance system provides lipid profile check-ups for those aged over 40, but the majority of our patients were younger than 40. Therefore, not all patients had laboratory data on sugar and lipid levels for further investigation on the association of metabolic syndrome and IBD. Therefore, TE with the controlled attenuation parameter technology is a relatively new method for the assessment of IBD, and no valid criteria for the classification of steatosis and fibrosis specific for these patients have been established [11, 14]. Hence, larger-scale and longer-term follow-up studies should be conducted to evaluate the use of such a technique in patients with IBD.

## Conclusions

In this study, we report the first result of NAFLD prevalence in an Asian population utilizing controlled attenuation parameter technology. The prevalence is currently low compared in our population with the Western population. Higher BMI and elder age are associated with the presence of NAFLD in our study.

## Supporting information

S1 Data (CSV)

## References

[1] Yen HH, Chen MW, Chang YY, Huang HY, Hsu TC, Chen YY. Predictive values of stool-based tests for mucosal healing among Taiwanese patients with ulcerative colitis: a retrospective cohort analysis. PeerJ. 2020;8:e9537. Epub 2020/08/04. 10.7717/peerj.9537 32742803 PMC7367046

[2] Yen HH, Weng MT, Tung CC, Wang YT, Chang YT, Chang CH, et al. Epidemiological trend in inflammatory bowel disease in Taiwan from 2001 to 2015: a nationwide populationbased study. Intest Res. 2019;17(1):54–62. Epub 2018/11/20. 10.5217/ir.2018.00096 30449079 PMC6361021

[3] Wei SC, Chang TA, Chao TH, Chen JS, Chou JW, Chou YH, et al. Management of ulcerative colitis in Taiwan: consensus guideline of the Taiwan Society of Inflammatory Bowel Disease. Intest Res. 2017;15(3):266–84. Epub 2017/07/04. 10.5217/ir.2017.15.3.266 28670225 PMC5478753

[4] Wei SC, Chang TA, Chao TH, Chen JS, Chou JW, Chou YH, et al. Management of Crohn’s disease in Taiwan: consensus guideline of the Taiwan Society of Inflammatory Bowel Disease. Intest Res. 2017;15(3):285–310. Epub 2017/07/04. 10.5217/ir.2017.15.3.285 28670226 PMC5478754

[5] Tien YC, Yen HH, Chiu YM. Incidence and clinical characteristics of hepatitis B virus reactivation in HBsAg-negative/HBcAb-positive patients receiving rituximab for rheumatoid arthritis. Clin Exp Rheumatol. 2017;35(5):831–6. Epub 2017/04/05. . 28375829

[6] Yen HH, Su PY, Zeng YH, Liu IL, Huang SP, Hsu YC, et al. Glecaprevir-pibrentasvir for chronic hepatitis C: Comparing treatment effect in patients with and without end-stage renal disease in a real-world setting. PLoS One. 2020;15(8):e0237582. Epub 2020/08/14. 10.1371/journal.pone.0237582 32790715 PMC7425913

[7] Li J, Zou B, Yeo YH, Feng Y, Xie X, Lee DH, et al. Prevalence, incidence, and outcome of non-alcoholic fatty liver disease in Asia, 1999–2019: a systematic review and meta-analysis. Lancet Gastroenterol Hepatol. 2019;4(5):389–98. Epub 2019/03/25. 10.1016/S2468-1253(19)30039-1 . 30902670

[8] McGowan CE, Jones P, Long MD, Barritt ASt. Changing shape of disease: nonalcoholic fatty liver disease in Crohn’s disease-a case series and review of the literature. Inflamm Bowel Dis. 2012;18(1):49–54. Epub 2011/02/26. 10.1002/ibd.21669 21351214 PMC3137748

[9] Lin A, Roth H, Anyane-Yeboa A, Rubin DT, Paul S. Prevalence of Nonalcoholic Fatty Liver Disease in Patients With Inflammatory Bowel Disease: A Systematic Review and Meta-analysis. Inflamm Bowel Dis. 2020. Epub 2020/08/12. 10.1093/ibd/izaa189 . 32780094 PMC8600033

[10] Karaivazoglou K, Konstantakis C, Tourkochristou E, Assimakopoulos SF, Triantos C. Non-alcoholic fatty liver disease in inflammatory bowel disease patients. Eur J Gastroenterol Hepatol. 2020. Epub 2020/02/12. 10.1097/MEG.0000000000001679 . 32044821

[11] Saroli Palumbo C, Restellini S, Chao CY, Aruljothy A, Lemieux C, Wild G, et al. Screening for Nonalcoholic Fatty Liver Disease in Inflammatory Bowel Diseases: A Cohort Study Using Transient Elastography. Inflamm Bowel Dis. 2019;25(1):124–33. Epub 2018/06/12. 10.1093/ibd/izy200 . 29889226

[12] Sagami S, Ueno Y, Tanaka S, Fujita A, Hayashi R, Oka S, et al. Significance of non-alcoholic fatty liver disease in Crohn’s disease: A retrospective cohort study. Hepatol Res. 2017;47(9):872–81. Epub 2016/10/14. 10.1111/hepr.12828 . 27737498

[13] Arieira C, Monteiro S, Xavier S, Dias de Castro F, Magalhaes J, Moreira MJ, et al. Hepatic steatosis and patients with inflammatory bowel disease: when transient elastography makes the difference. Eur J Gastroenterol Hepatol. 2019;31(8):998–1003. Epub 2019/03/07. 10.1097/MEG.0000000000001319 . 30839437

[14] Lin YJ, Lin CH, Wang ST, Lin SY, Chang SS. Noninvasive and Convenient Screening of Metabolic Syndrome Using the Controlled Attenuation Parameter Technology: An Evaluation Based on Self-Paid Health Examination Participants. J Clin Med. 2019;8(11). Epub 2019/10/28. 10.3390/jcm8111775 31653028 PMC6912761

[15] Yen YH, Kuo FY, Lin CC, Chen CL, Chang KC, Tsai MC, et al. Predicting Hepatic Steatosis in Living Liver Donors Via Controlled Attenuation Parameter. Transplant Proc. 2018;50(10):3533–8. Epub 2018/12/24. 10.1016/j.transproceed.2018.06.039 . 30577232

[16] Yen YH, Chen JB, Cheng BC, Chen JF, Chang KC, Tseng PL, et al. Using controlled attenuation parameter combined with ultrasound to survey non-alcoholic fatty liver disease in hemodialysis patients: A prospective cohort study. PLoS One. 2017;12(4):e0176027. Epub 2017/04/21. 10.1371/journal.pone.0176027 28426815 PMC5398606

[17] Karlas T, Petroff D, Sasso M, Fan JG, Mi YQ, de Ledinghen V, et al. Individual patient data meta-analysis of controlled attenuation parameter (CAP) technology for assessing steatosis. J Hepatol. 2017;66(5):1022–30. Epub 2017/01/01. 10.1016/j.jhep.2016.12.022 . 28039099

[18] Wong VW, Vergniol J, Wong GL, Foucher J, Chan HL, Le Bail B, et al. Diagnosis of fibrosis and cirrhosis using liver stiffness measurement in nonalcoholic fatty liver disease. Hepatology. 2010;51(2):454–62. Epub 2010/01/27. 10.1002/hep.23312 . 20101745

[19] Dasarathy S, Dasarathy J, Khiyami A, Joseph R, Lopez R, McCullough AJ. Validity of real time ultrasound in the diagnosis of hepatic steatosis: a prospective study. J Hepatol. 2009;51(6):1061–7. Epub 2009/10/23. 10.1016/j.jhep.2009.09.001 19846234 PMC6136148

[20] Zou ZY, Shen B, Fan JG. Systematic Review With Meta-analysis: Epidemiology of Nonalcoholic Fatty Liver Disease in Patients With Inflammatory Bowel Disease. Inflamm Bowel Dis. 2019;25(11):1764–72. Epub 2019/03/29. 10.1093/ibd/izz043 . 30918952

[21] Ritaccio G, Stoleru G, Abutaleb A, Cross RK, Shetty K, Sakiani S, et al. Nonalcoholic Fatty Liver Disease Is Common in IBD Patients However Progression to Hepatic Fibrosis by Noninvasive Markers Is Rare. Dig Dis Sci. 2020. Epub 2020/09/08. 10.1007/s10620-020-06588-6 . 32894439 PMC7936981

[22] Kang MK, Kim KO, Kim MC, Park JG, Jang BI. Sarcopenia is a new risk factor of non-alcoholic fatty liver disease in patients with inflammatory bowel disease. Dig Dis. 2020. Epub 2020/03/07. 10.1159/000506938 . 32135539

[23] Yen H SP, Liu I, Zeng Y, Huang S, Hsu Y, Yang C, et al. Direct-acting antiviral treatment for Hepatitis C Virus in geriatric patients: a real-world retrospective comparison between early and late elderly patients. PeerJ. 2021;9:e10944. 10.7717/peerj.10944 33777520 PMC7977377

[24] Yen HH, Su PY, Liu II, Zeng YH, Huang SP, Hsu YC, et al. Retrieval of lost patients in the system for hepatitis C microelimination: a single-center retrospective study. BMC Gastroenterol. 2021;21(1):209. Epub 2021/05/10. 10.1186/s12876-021-01792-8 . 33964873 PMC8105932

[25] Zhou F, Zhou J, Wang W, Zhang XJ, Ji YX, Zhang P, et al. Unexpected Rapid Increase in the Burden of NAFLD in China From 2008 to 2018: A Systematic Review and Meta-Analysis. Hepatology. 2019;70(4):1119–33. Epub 2019/05/10. 10.1002/hep.30702 . 31070259

[26] Chalasani N, Younossi Z, Lavine JE, Charlton M, Cusi K, Rinella M, et al. The diagnosis and management of nonalcoholic fatty liver disease: Practice guidance from the American Association for the Study of Liver Diseases. Hepatology. 2018;67(1):328–57. Epub 2017/07/18. 10.1002/hep.29367 . 28714183

[27] Younossi ZM, Koenig AB, Abdelatif D, Fazel Y, Henry L, Wymer M. Global epidemiology of nonalcoholic fatty liver disease-Meta-analytic assessment of prevalence, incidence, and outcomes. Hepatology. 2016;64(1):73–84. Epub 2015/12/29. 10.1002/hep.28431 . 26707365

[28] Ciccone MM, Principi M, Ierardi E, Di Leo A, Ricci G, Carbonara S, et al. Inflammatory bowel disease, liver diseases and endothelial function: is there a linkage? J Cardiovasc Med (Hagerstown). 2015;16(1):11–21. Epub 2014/11/27. 10.2459/JCM.0000000000000149 . 25427048

[29] Lin WC, Weng MT, Tung CC, Chang YT, Leong YL, Wang YT, et al. Trends and risk factors of mortality analysis in patients with inflammatory bowel disease: a Taiwanese nationwide population-based study. J Transl Med. 2019;17(1):414. Epub 2019/12/14. 10.1186/s12967-019-02164-3 31831015 PMC6909461

[30] Chao CY, Battat R, Al Khoury A, Restellini S, Sebastiani G, Bessissow T. Co-existence of non-alcoholic fatty liver disease and inflammatory bowel disease: A review article. World J Gastroenterol. 2016;22(34):7727–34. Epub 2016/09/30. 10.3748/wjg.v22.i34.7727 27678354 PMC5016371

[31] Cheon JH. Genetics of inflammatory bowel diseases: a comparison between Western and Eastern perspectives. J Gastroenterol Hepatol. 2013;28(2):220–6. Epub 2012/11/30. 10.1111/jgh.12053 . 23189979

[32] Mak WY, Zhao M, Ng SC, Burisch J. The epidemiology of inflammatory bowel disease: East meets west. J Gastroenterol Hepatol. 2020;35(3):380–9. Epub 2019/10/10. 10.1111/jgh.14872 . 31596960

[33] Maaser C, Sturm A, Vavricka SR, Kucharzik T, Fiorino G, Annese V, et al. ECCO-ESGAR Guideline for Diagnostic Assessment in IBD Part 1: Initial diagnosis, monitoring of known IBD, detection of complications. J Crohns Colitis. 2019;13(2):144–64. Epub 2018/08/24. 10.1093/ecco-jcc/jjy113 . 30137275

[34] Adams LC, Lubbe F, Bressem K, Wagner M, Hamm B, Makowski MR. Non-alcoholic fatty liver disease in underweight patients with inflammatory bowel disease: A case-control study. PLoS One. 2018;13(11):e0206450. 10.1371/journal.pone.0206450 30427909 PMC6241122

[35] Amorim VB, Parente DB, Paiva FF, Oliveira Neto JA, Miranda AA, Moreira CC, et al. Can gadoxetic acid-enhanced magnetic resonance imaging be used to avoid liver biopsy in patients with nonalcoholic fatty liver disease? World J Hepatol. 2020;12(9):661–71. Epub 2020/10/10. 10.4254/wjh.v12.i9.661 33033571 PMC7522564

[36] Barbero-Villares A, Mendoza Jimenez-Ridruejo J, Taxonera C, Lopez-Sanroman A, Pajares R, Bermejo F, et al. Evaluation of liver fibrosis by transient elastography (Fibroscan(R)) in patients with inflammatory bowel disease treated with methotrexate: a multicentric trial. Scand J Gastroenterol. 2012;47(5):575–9. Epub 2012/01/11. 10.3109/00365521.2011.647412 . 22229701

[37] Allocca M, Danese S, Laurent V, Peyrin-Biroulet L. Use of Cross-Sectional Imaging for Tight Monitoring of Inflammatory Bowel Diseases. Clin Gastroenterol Hepatol. 2020;18(6):1309–23 e4. Epub 2019/12/10. 10.1016/j.cgh.2019.11.052 . 31812657

[38] Yen HH, Hsu TC, Chen MW, Su PY, Chen YY. Clinical features and treatment of inflammatory bowel disease in a low-incidence area: A hospital-based retrospective cohort study in Taiwan. Medicine (Baltimore). 2021;100(10):e25090. Epub 2021/03/18. 10.1097/MD.0000000000025090 33725901 PMC7969237

[39] Estes C, Chan HLY, Chien RN, Chuang WL, Fung J, Goh GB, et al. Modelling NAFLD disease burden in four Asian regions-2019-2030. Aliment Pharmacol Ther. 2020;51(8):801–11. Epub 2020/03/07. 10.1111/apt.15673 32133676 PMC7154715

[40] Huang JF, Yeh ML, Yu ML, Huang CF, Dai CY, Hsieh MY, et al. Hyperuricemia Inversely Correlates with Disease Severity in Taiwanese Nonalcoholic Steatohepatitis Patients. PLoS One. 2015;10(10):e0139796. Epub 2015/10/07. 10.1371/journal.pone.0139796 26441244 PMC4595446

